# Targeting tumor angiogenesis and metabolism: a new perspective in pediatric thoracic tumor therapy

**DOI:** 10.3389/fcell.2025.1558403

**Published:** 2025-03-27

**Authors:** Yong Lv, Fanke Shu, Dengke Luo, Ru Jia, YiDong Huang, Chang Xu

**Affiliations:** Department of Pediatric Surgery, West China Hospital, Sichuan University, Chengdu, China

**Keywords:** pediatric, thoracic, tumor, angiogenesis, metabolism

## Abstract

Pediatric thoracic solid tumors encompass mediastinal tumors, chest wall tumors, and lung tumors. The pathogenesis is complex, and the clinical presentation is diverse, presenting numerous challenges in diagnosis and treatment, which severely threaten the life and health of the affected children. Angiogenesis provides nutritional and oxygen support for tumor growth and metastasis, while metabolic reprogramming meets the unique energy and material demands of tumor. Both processes play key roles in pediatric thoracic tumor development. Therefore, targeting tumor vasculature could be an important therapeutic strategy, and exploring the molecular mechanisms of metabolic reprogramming may provide a theoretical foundation for targeted treatment. This review summarizes relevant experimental research on angiogenesis and metabolic reprogramming in pediatric thoracic tumors, analyzes the limitations of current research, and proposes solutions and recommendations. Through this review, we aim to provide comprehensive information about pediatric thoracic solid tumors for clinicians and researchers, promoting personalized treatment, and ultimately improve survival rates and quality of life for affected children.

## 1 Introduction to pediatric thoracic tumor

**FIGURE 1:**
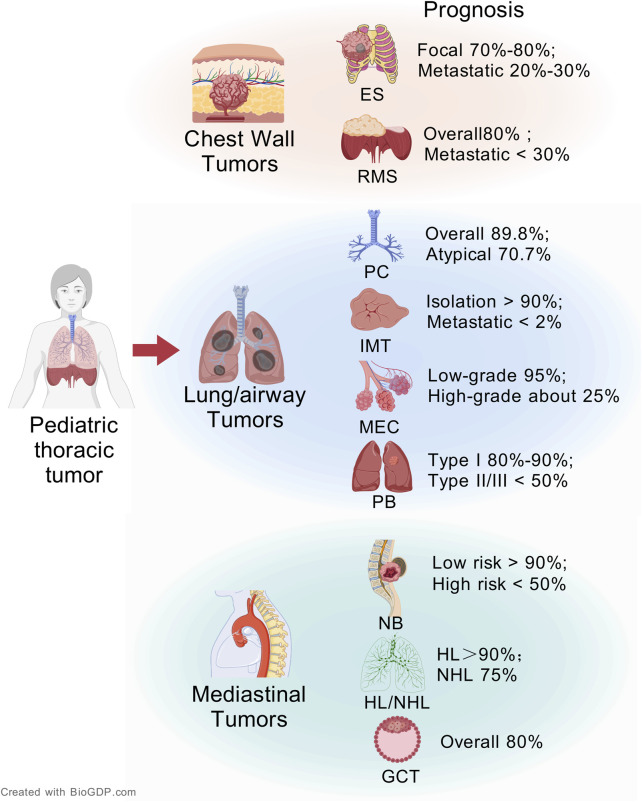
The overview of pediatric thoracic tumors and its prognosis (Created with BioGDP.com (Jiang et al., 2025)).

Pediatric thoracic tumors are a heterogeneous group of neoplasms, accounting for 15%–20% of all malignant tumors in childhood (Meazza and Gattuso, 2019). Pediatric thoracic tumor included tumors in areas such as the chest wall, lungs, airways, mediastinal organs, esophagus, and diaphragm (Zapala et al., 2017). They are primarily composed of abnormal tissue formed from residual embryonic tissue or metastatic tumors (Mullen et al., 2021). Clinically, neurogenic tumors and lymphomas are relatively common (Jaggers and Balsara, 2004). The insidious progression of many thoracic tumors frequently delays diagnosis, initial asymptomatic phases transition to clinically significant manifestations when mass effects compromise critical structures, such as airway compression precipitating stridor, mediastinal invasion causing superior vena cava syndrome, and diaphragmatic involvement leading to respiratory insufficiency (Guye et al., 2007). The incidence of malignant pediatric thoracic tumor has been an increasing trend in recent years (Bhakta et al., 2019). Pediatric thoracic tumors present a heterogeneous prognostic landscape (Figure 1). Many children with thoracic tumors have already progressed to the middle and late stages when they are diagnosed, which seriously affects the survival rate of the children, and has become one of the main causes of death in children (Lam et al., 2019).

### 1.1 Clinical treatment for pediatric thoracic tumor

Pediatric thoracic tumors are mainly divided into chest wall tumors, lung and airway tumors, and mediastinal tumors according to their origin. At present, the main treatment methods of pediatric thoracic tumors are still surgery and chemotherapy. We integrate the pathological features and clinical management of various pediatric thoracic tumors. Table 1 systematically summarizes the anatomic sites, origin cells, involved organs, recommended treatment options, and current treatment challenges of each pediatric thoracic tumor.

**TABLE 1:** Pediatric thoracic tumors: Classification and clinical characteristics.

Tumor type	Cellular origin/Molecular features	Affected organs/Systems	First-line therapies	Current limitations
Chest Wall Tumors				
Ewing Sarcoma (ES) (Wang et al., 2021) (van den Berg et al., 2008) (Riggi et al., 2021)	Bone/soft tissue progenitors; EWS-FLI1 fusion	Ribs, soft tissues	Surgical resection + chemotherapy (vincristine, actinomycin-D, cyclophosphamide); emerging targeted therapies	High mortality in metastatic cases; delayed clinical translation of fusion-targeted drugs
Rhabdomyosarcoma (RMS) (Chen et al., 2024a) (Zarrabi et al., 2023) (Mascarenhas et al., 2019)	Primitive mesenchymal cells; PAX3/7-FOXO1 fusion	Intercostal muscles	Neoadjuvant chemotherapy surgery ± radiotherapy	Molecular heterogeneity; aggressive behavior in PAX-fusion-positive subtypes
Lung/airway Tumors				
Inflammatory Myofibroblastic Tumor (IMT) (Casanova et al., 2020) (Chmiel et al., 2024; Mahajan et al., 2021)	Spindle cells; ALK rearrangement	Lungs, bronchi	Complete excision; ALK inhibitors (e.g., crizotinib)	Limited chemosensitivity in unresectable cases
Pulmonary Carcinoid (PC) (Petursdottir et al., 2020) (Uprety et al., 2020)	Neuroendocrine cells	Bronchial tree	Surgical resection ± somatostatin analogs; etoposide/cisplatin for advanced cases	Metastasis risk in atypical variants; lack of optimized chemotherapy regimens
Mucoepidermoid Carcinoma (MEC) (Horio et al., 2024) (Wu et al., 2019; Li et al., 2017)	Airway mucous glands; CRTC1-MAML2 fusion	Tracheobronchial system	Surgical resection; EGFR inhibitors (e.g., gefitinib)	Controversy over adjuvant therapy for high-grade tumors; unclear mechanisms of EGFR targeting
Pleuropulmonary Blastoma (PPB) (Thacker et al., 2023) (Dehner et al., 2019) (Knight et al., 2019)	DICER1 mutation	Lungs, pleura	Surgery ± chemoradiotherapy	Metastasis in 30% of Type II/III; limited availability of DICER1 mutation screening
Mediastinal Tumors				
Neuroblastoma (NB) (Zheng et al., 2024) (Liang et al., 2020) (Wahba et al., 2023)	Neural crest cells; MYCN amplification	Posterior mediastinum	Surgery + chemotherapy; anti-GD2 immunotherapy; ALK/MEK inhibitors (e.g., crizotinib)	Therapy resistance in high-risk group; slow clinical translation of targeted agents
Lymphoma (HL/NHL) (Bucklein, 2023) (Metzger and Mauz-Korholz, 2019; Mauz-Korholz et al., 2018)	B/T lymphocytes	Anterior mediastinum	Chemotherapy ± low-dose RT; anti-CD30/CD20 antibodies (e.g., rituximab)	Requirement of stem cell transplantation in refractory cases; radiation-related toxicities
Germ Cell Tumor (GCT) (Mosbech et al., 2014) (De Felici et al., 2021) (Hulsker et al., 2021)	Primordial germ cells	Anterior mediastinum	Surgery ± cisplatin-based chemotherapy; TKIs (e.g., sunitinib)	Poor prognosis of primary mediastinal GCT; insufficient evidence for targeted therapies

Annotation. HL: Hodgkin Lymphoma; NHL: Non-Hodgkin Lymphoma; GCT: Germ Cell Tumor; ALK: Anaplastic Lymphoma Kinase; EGFR: Epidermal Growth Factor Receptor; GD2: Disialoganglioside GD2; MEK: Mitogen-activated protein kinase; TKIs: Tyrosine Kinase Inhibitors; PAX3/7: Paired Box gene 3/7; FOXO1: Forkhead Box O1; CRTC1: CREB Regulated Transcription Coactivator 1; MAML2: Mastermind-Like Protein 2; DICER1: Ribonuclease III enzyme DICER1.

### 1.2 Critical differences between pediatric thoracic tumor and adult cancers

Pediatric thoracic tumors in children differ significantly from those in adults in terms of pathogenesis and genetic landscape (Chen X. et al., 2024). Firstly, the types of tumors are different. Pediatric thoracic tumors often arise from developmental tissues and are associated with genetic mutations present at birth or acquired early in life, whereas adult thoracic malignancies are primarily driven by lifestyle-induced somatic mutations (Kattner et al., 2019). Secondly, there are physiological differences between children and adults. For example, pediatric chest cavity is smaller, and compared to adults, thoracic tumors are more likely to compress the heart, airways, and esophagus, leading to respiratory and circulatory functional disorders (Pearson and Tan, 2015). Furthermore, pediatric organs are not fully mature, and pediatric medications carry higher risks, chemotherapy drugs at adult doses can be highly toxic to children (Weingart et al., 2018). Given the specific physiological, pathological, and pharmacokinetic differences in children, there is an urgent need to develop treatment specifically for pediatric thoracic tumors to improve therapeutic efficacy.

### 1.3 Challenges in pediatric thoracic tumor treatment

Pediatric thoracic tumors demonstrate aggressive growth patterns with frequent thoracic occupation, inducing severe compression or invasion of cardiopulmonary-vascular structures (Sandler and Hayes-Jordan, 2018). While surgery and chemotherapy remain cornerstone therapies, their efficacy diminishes significantly in recurrent or refractory cases (Burkhardt and Hermiston, 2019). Although targeted therapies against angiogenesis and tumor metabolism pathways hold transformative potential, clinical translation is hampered by insufficient pediatric-specific drug development and trial data (Dasgupta et al., 2017). This review will summarize the current research progress on targeting angiogenesis and tumor cell metabolism, and discuss their potential applications in the treatment of pediatric thoracic tumors, with the aim of providing new insights and directions for future treatment strategies.

## 2 Targeted tumor vascularization strategies in pediatric thoracic tumors

### 2.1 Anti-angiogenesis

Angiogenesis is a critical step in tumor growth and metastasis, serving to transport nutrients and remove metabolic waste products from tumor cells (Jiang et al., 2020). Tumors induce angiogenesis to promote metastasis and proliferation, inhibiting pro-angiogenic factors, such as vascular endothelial growth factor (VEGF), is a key anti-angiogenic therapy (Janes et al., 2024). Bevacizumab, a VEGF-specific antibody, was the first clinically approved anti-angiogenic drug (Garcia et al., 2020). Research data indicate that low doses of bevacizumab not only reduce the formation of pathological blood vessels but also repair existing vascular defects (Summers et al., 2010). This further demonstrates that appropriate doses of VEGF inhibitors can rebalance angiogenesis signaling within tumors, actively recruit pericytes, and enhance intercellular junctions (Jain et al., 2006). Researchers have found that bevacizumab treatment reduced microvessel density in alveolar RMS, increased tumor vessel maturity, enhanced the effectiveness of ionizing radiation for alveolar RMS, and improved patient prognosis (Myers et al., 2010). However, due to the relative specificity of these targets, their clinical application still faces some challenges. Research evidence suggests that anti-VEGF therapy may enhance the signaling of platelet-derived growth factor receptor, thereby promoting the recruitment of pericytes and reducing vascular permeability. The high coverage of pericytes supports the survival of tumor endothelial cells, and the hypoxic environment caused by the disruption of the tumor vasculature further stimulates tumor cells to develop stronger resistance and enhanced angiogenic capability (Greenberg et al., 2008). Therefore, new VEGF-targeted therapies are still under development. Sunitinib is a novel multi-targeted inhibitor, and multiple clinical trials have demonstrated its efficacy in patients with solid tumors. Sunitinib inhibits several tyrosine kinases, particularly the VEGFR, thereby blocking the angiogenesis process required for tumor growth, resulting in clinical benefits for patients with mediastinal germ cell tumors (Subbiah et al., 2014). Primary mediastinal germ cell tumors exhibit significant angiogenic characteristics, with the formation of abnormal tumor blood vessels in the incompletely matured stroma. These blood vessels may contribute to a certain degree of resistance to treatment (Levy et al., 2021). Researchers used CRISPR/Cas9 technology to knock out the REST gene in Ewing sarcoma cells. *In vivo* experiments revealed that REST knockout tumors showed a significant reduction in pericytes and blood vessel perfusion, with a marked decrease in the ratio of pericytes to endothelial cells, increased vascular leakage, and induced tumor hypoxic, leading to inhibition of tumor growth and metastasis (Zhou et al., 2020). These findings confirm the clinical potential of vascular target therapy in Ewing sarcoma.

Additional studies targeting anti-angiogenesis have focused on angiogenesis-related signal pathways. The FGF/FGFR signaling pathway also plays an important role in promoting tumor angiogenesis, and FGF is a pro-angiogenic factor that synergistically induces tumor angiogenesis with VEGF (Presta et al., 2017). Researchers found that the FGF pathway and neural cell adhesion molecule 1 (NCAM1) were activated in the gene expression profile of pleuropulmonary blastoma, and that intervening in the FGF signaling pathway by targeting NCAM1 could effectively inhibit tumor growth and progression, which provided a new idea for pleuropulmonary blastoma treatment (Shukrun et al., 2019). TGF-β has been shown to stimulate angiogenesis, and the abundant vasculature in carcinoid may be a result of the indirect effects of TGF-β within the tumor stroma. The role of TGF-β in pulmonary carcinoid depends on its microenvironment, and modulating TGF-β could become one of the strategies for targeted therapy in pulmonary carcinoid (Slodkowska et al., 2000). Activation of the EGFR signaling pathway induces upregulation of VEGF expression to drive tumor growth (Hung et al., 2016). The high-grade mucoepidermoid carcinomas exhibit stronger invasiveness. In high-grade MECs, the EGFR and ERK signaling pathways are often abnormally activated, targeting EGFR may provide additional therapeutic benefits, potentially improving patient survival prognosis and quality of life (Lujan et al., 2010).

### 2.2 Vascular normalization

Due to the excessive and sustained release of pro-angiogenic factors within the tumor microenvironment, the newly formed vascular network may fail to mature. The uneven caliber of blood vessels and the tortuous, disorganized structure of the vascular network subsequently contribute to increased localized hypoxia (Guelfi et al., 2024). Furthermore, the reduced density of pericytes and their decreased connection to endothelial cells lead to abnormal tumor vasculature. The characteristics of these abnormal blood vessels, including high permeability and low perfusion, impair drug delivery and thereby decrease the effectiveness of tumor treatment (Jiang Z. et al., 2023). In 2001, the concept of vascular normalization was formally introduced, which involves remodeling tumor blood vessels to restore their structure and function to improve vascular oxygenation and perfusion, rather than solely disrupting the growth of blood vessels (Jaszai and Schmidt, 2019). In the context of pediatric thoracic tumors, vascular normalization holds unique promise. Studies in pediatric thoracic tumor models, such as alveolar rhabdomyosarcoma, demonstrate that mTOR/VEGF inhibition (e.g., rapamycin) can normalize vasculature and enhance radiation efficacy (Myers et al., 2012). Studies in pediatric tumor neuroblastoma, demonstrate that anlotinib can normalize vasculature and induces tumor regression (Su et al., 2022). However, clinical translation remains limited, with few trials explicitly testing vascular normalization strategies in pediatric thoracic tumors. While anti-angiogenic drugs (e.g., VEGF inhibitors) reduce tumor vascularity, they may exacerbate hypoxia and promote treatment resistance in slow-growing pediatric tumors, which often rely on prolonged therapy (Lupo et al., 2016). Vascular normalization, by contrast, aims to stabilize the tumor microenvironment, potentially improving chemotherapy or radiation sensitivity and reducing metastatic shedding (Yu et al., 2023), a critical advantage for children with thoracic tumor. The scarcity of dedicated studies in pediatric thoracic tumors reflects challenges in modeling these rare cancers and concerns about long-term vascular toxicity in developing tissues. Nevertheless, emerging data from adult thoracic malignancies (e.g., lung cancer) and pediatric preclinical models suggest that normalization could mitigate hypoxia-driven aggression and therapy resistance in these tumors, justifying its inclusion in this review (Jiang S. et al., 2023).

### 2.3 Immune cells are involved in regulating tumor angiogenesis

Immune cells can coordinate the entire process of tumor angiogenesis. Innate immune cells, such as mature dendritic cells and M1 tumor-associated macrophages, produce cytokines that inhibit tumor angiogenesis. Adaptive immune cells, such as CD8^+^ T cells and T helper cells 1, secrete interferons, which are effective cytokines that suppress angiogenesis and induce vascular normalization (Delprat and Michiels, 2021). Immune cells play a complex role in tumor angiogenesis, and combining immune cell therapy and targeting tumor vasculature can help to personalize tumor therapy. Ewing sarcoma is a highly vascularized tumor with consistent expression of VEGFR2 on its blood vessels. Chimeric Antigen Receptor (CAR) T-cell engineering directly links T cells to specific tumor markers, enhancing their ability to recognize and kill tumor cells. Researchers used VEGFR2-specific CAR-T cells to selectively target and disrupt the Ewing sarcoma tumor-associated vasculature, inducing hypoxic tumor cell death and eradicating the tumor (Englisch et al., 2020). Neuroblastoma is characterized by a highly vascularized nature. Studies have shown that Anlotinib, by inhibiting pro-angiogenic factor receptors and reducing pro-angiogenic factors, reverses the early exhaustion of CD4^+^ T cells. This promotes tumor vascular normalization, leading to increased infiltration of immune effector cells and significantly inducing the regression of neuroblastoma (Su et al., 2022).

## 3 Strategies for targeting tumor metabolism in pediatric thoracic tumors

Tumor cells reprogram their metabolism to meet their energy demands, characterized by upregulation of aerobic glycolysis, increased fatty acid synthesis, and enhanced amino acid catabolism. As one of the important markers in the process of malignant tumor occurrence and development, metabolic reprogramming provides an important material and energy basis for tumor cell proliferation and metastasis, in which glucose metabolism, fatty acid metabolism and amino acid metabolism play important roles (Xiang et al., 2023). Metabolic reprogramming not only acts on tumor cells but also regulates tumor by influencing the tumor microenvironment (Zhang et al., 2024). Therefore, targeted therapies based on tumor metabolism have gained widespread attention. Targeted drugs developed for glucose metabolism, lipid metabolism, and amino acid metabolism have shown promising results in both basic research and clinical trials (Wang et al., 2024). Then we provide a systematic exposition of metabolic reprogramming in pediatric thoracic solid tumors, and this may help in recognizing tumor vulnerabilities and identifying new therapeutic targets.

### 3.1 Glycolysis and oxidative phosphorylation

Glycolysis and mitochondrial oxidative phosphorylation are two main pathways of cellular energy metabolism, and their roles in pediatric thoracic solid tumors are complex and diverse. Glycolysis is a process in the cytoplasm where glucose is broken down into pyruvate, and even under aerobic conditions, tumor cells tend to generate energy through glycolysis, a phenomenon known as the Warburg effect. The key enzymes in the glycolytic process, such as hexokinase, phosphofructokinase, and pyruvate kinase, have become research targets for cancer therapy (Tufail et al., 2024). Mitochondrial oxidative phosphorylation is a process that generates ATP through the electron transport chain and the tricarboxylic acid cycle. Mitochondrial oxidative phosphorylation not only provides energy to the cell, but also participates in cellular anabolism and signal transduction, which is essential for the proliferation and survival of tumor cells (Wu et al., 2022). Studying the role of glycolysis and mitochondrial oxidative phosphorylation not only provides clues for understanding metabolic reprogramming in pediatric thoracic tumors, but also offers potential targets for developing new therapeutic strategies. The metabolism of Ewing sarcoma cells relies on aerobic glycolysis. Melatonin reduces glycolytic metabolism by inhibiting the activity of HIF-1α in Ewing sarcoma cells, leading to decreased glucose uptake, lactate levels, and lactate dehydrogenase activity. This effectively suppresses the growth and survival of Ewing sarcoma cells, with potential therapeutic value (Sanchez-Sanchez et al., 2015). MYCN proto-oncogene (MYCN) acts as both an oncogenic driver and a metabolic reprogramming regulator, altering cellular energy metabolism to fuel neuroblastoma progression (Talapatra and Reddy, 2023). Research has shown that MYCN amplification can significantly enhance the uptake and consumption of glucose in neuroblastoma. Specifically, MYCN, as an oncogenic protein, directly regulates several key enzymes involved in glucose metabolism, including enzymes in the glycolytic pathway, thereby promoting the elevation of glycolysis (Oliynyk et al., 2019). Considering this metabolic characteristic, glycolysis inhibitors have been used to treat MYCN-amplified neuroblastoma. These glycolysis inhibitors effectively inhibit glycolytic activity in MYCN-amplified cells in neuroblastoma models, thereby reducing the energy supply to tumor cells and limiting their proliferative capacity, demonstrating potential antitumor effects (Levy et al., 2012; Hagenbuchner et al., 2016). MYCN also regulates mitochondrial respiration and oxidative phosphorylation by increasing the expression of various enzymes in neuroblastoma, thereby supporting the rapid proliferation of tumor cells. In this context, depletion of dihydrolipoamide succinyltransferase can significantly inhibit the production of reduced nicotinamide adenine dinucleotide, disrupt oxidative phosphorylation, and subsequently impair neuroblastoma formation and inhibit tumor invasion (Anderson et al., 2021). Moreover, the combination therapy of vorinostat and rapamycin significantly reduced levels of two critical glycolysis rate-limiting enzymes - Hexokinase 2 (HK2, catalyzing the first phosphorylation step) and Glucose-6-phosphate isomerase (GPI, mediating glucose-6-phosphate isomerization) - in neuroblastoma models. This suppression coincided with elevated reactive oxygen species (ROS) levels, effectively blocking tumor metabolic reprogramming and revealing therapeutic potential for advanced neuroblastoma (Bishayee et al., 2022).

### 3.2 Amino acid and nucleic acid metabolism

Glutamine is a major amino acid in the body and a primary fuel source for tumor cells (Jain et al., 2012). The consumption of glutamine disrupts the redox equilibrium of tumor cells, thereby decreasing the proliferation capacity of neuroblastoma cells and enhancing the radiosensitivity of non-MYCN amplified tumor cell. Targeting glutamine metabolism might be a potential therapeutic approach for neuroblastoma (Le Grand et al., 2020). Research has found that rhabdomyosarcoma consumes more glucose and glutamine than healthy tissue. After receiving radiation therapy, the rhabdomyosarcoma undergoes metabolic reprogramming, characterized by a weakened tricarboxylic acid cycle involving glucose and a shift towards reactions dominated by glutamine metabolism. Inhibiting glutaminase *in vivo* can enhance the effectiveness of tumor radiation therapy, suggesting that drug targeting of glutamine metabolism could be a method to sensitize rhabdomyosarcoma patients to radiotherapy (Patel et al., 2024). One-carbon metabolism is a series of biochemical reactions that involve the transfer, oxidation, or reduction of single-carbon units. These units are crucial for the synthesis of essential biomolecules like nucleotides, certain amino acids, and other cellular components. The major pathways of one-carbon metabolism include serine metabolism, glycine metabolism, and folate metabolism (Yang et al., 2021). Researchers have discovered that the Serine-glycine-one-carbon (SGOC) biosynthetic pathway is significantly activated in MYCN amplified neuroblastoma cells. Tumor cells utilize the SGOC pathway to convert carbon sources from glucose into serine and glycine to meet their needs for rapid proliferation. Based on this finding, researchers have used small molecule inhibitors to effectively block the SGOC pathway, not only disrupting the amino acid synthesis but also enhancing metabolic stress, thereby promoting tumor cell death through autophagy (Xia et al., 2019). Methylenetetrahydrofolate dehydrogenase 1 (MTHFD1) is a key enzyme in the folate metabolic pathway, involved in one-carbon metabolism, and supports tumor cell growth by maintaining the redox homeostasis of NADPH. In neuroblastoma, by targeting and inhibiting the activity of MTHFD1, the folate metabolic pathway is further suppressed, increasing the level of ROS within tumor cells, enhancing their metabolic stress, and triggering tumor cell apoptosis, thereby exerting an anti-tumor effect (Guan et al., 2024). MYCN enhances the dependency of neuroblastoma cells on pyrimidine nucleotide synthesis by upregulating enzymes related to pyrimidine biosynthesis, such as dihydroorotate dehydrogenase (DHODH). Considering this metabolic characteristic, researchers have inhibited tumor progression by genetically editing or pharmacologically inhibiting DHODH. Additionally, using dipyridamole to inhibit nucleotide transport, in combination with DHODH inhibitors, has augmented the therapeutic efficacy against neuroblastoma (Yu et al., 2021). The EWS/FLI fusion gene (EF) drives metabolic reprogramming, diverting glycolytic intermediates towards the synthesis of serine and glycine to support the occurrence and development of Ewing sarcoma. Key regulatory enzymes in these metabolic reprogramming processes may become new therapeutic targets (Tanner et al., 2017).

### 3.3 Lipid metabolism

Lipids are one of the three major nutrients essential for maintaining life functions, energy storage, and metabolic homeostasis (Snaebjornsson et al., 2020). Lipid metabolism reprogramming is a significant metabolic characteristic of tumor, referring to the process where tumor cells readjust their lipid metabolic pathways in response to various internal and external stimuli. This adaptation promotes tumor to cope with new physiological conditions and sustains its rapid growth (Broadfield et al., 2021). In normal cells, the majority of lipids required for metabolism are obtained through dietary intake or synthesized in the liver. However, in tumor cells, lipid metabolism reprogramming favors *de novo* lipid synthesis, with lipids being stored inside the cells in the form of lipid droplets, and enriched in hypoxic tissue (Cao, 2019; Shakya et al., 2021). Citrate located in the cytosol is transformed into acetyl-CoA through the catalysis of ATP-citrate lyase. Subsequently, under the influence of acetyl-CoA carboxylase (ACC) and fatty acid synthase (FASN), acetyl-CoA is utilized for the synthesis of palmitic acid. Furthermore, stearoyl-CoA desaturase 1 (SCD1), long-chain fatty acid elongases, and fatty acid desaturase two are capable of further modifying and elongating palmitic acid, thereby generating fatty acids with diverse chain lengths and degrees of saturation (Jeon et al., 2023). Fatty acids can be oxidized and decomposed under the catalysis of a series of enzymes, generating energy, with β - oxidation being the principal catabolic pathway, carnitine palmitoyltransferase 1 (CPT1), a crucial rate-limiting enzyme in fatty acid β-oxidation (Ngo et al., 2023). Malonyl - CoA decarboxylase (MCD) is an important enzyme in cellular fatty acid oxidation (Ussher et al., 2016). Clinically actionable targets in this rewired metabolism include:

FASN: As a key enzyme catalyzing *de novo* fatty acid synthesis, FASN is activated in pediatric osteosarcoma with lung metastasis, suggesting that FASN is an important target for pediatric thoracic solid tumors (Liu et al., 2012).

ACC: The ACC is also a rate-limiting enzyme for fatty acid synthesis. Targeted inhibition of ACC will block fatty acid synthesis, thereby promoting neuroblastoma differentiation and reducing tumor load (Ruiz-Perez et al., 2021).

CPT1: In neuroblastoma, the suppression of CPT1, a crucial rate-limiting enzyme in fatty acid β-oxidation, results in a reduced extent of fatty acid β-oxidation and subsequently inhibits tumor growth (Oliynyk et al., 2019).

MCD: In rhabdomyosarcoma models, pharmacological inhibition of malonyl-CoA decarboxylase disrupted this balance by elevating malonyl-CoA levels and suppressing fatty acid oxidation, ultimately arresting cell cycle progression and impairing proliferation (Miyagaki et al., 2021).

## 4 Discussion

Pediatric thoracic solid tumors should be treated with a multidisciplinary approach. Targeting tumor blood vessels has emerged as an important strategy in tumor treatment, aimed at improving tumor blood supply, enhancing drug delivery, and increasing therapeutic effectiveness. However, targeting tumor vasculature still faces several challenges. One of the primary difficulties arises from the heterogeneous nature of tumor blood vessels. Tumors contain various cell types and tissues, which results in blood vessels with different morphological and functional characteristics across different regions of the tumor. This makes strategies to normalize tumor blood vessels more complex. The tumor microenvironment is composed of diverse cell types, including tumor cells, immune cells, and fibroblasts, which interact with the blood vessels in complex ways. These cells can release a variety of factors that influence the process of blood vessel normalization. Despite these challenges, ongoing research aims to develop more effective strategies to normalize tumor blood vessels and improve the delivery of tumor treatments. By better understanding the tumor vasculature and its interactions with the surrounding cells, scientists hope to overcome these obstacles and enhance the efficacy of vascular-targeted therapies. Tumor vascular normalization has emerged as a promising strategy to enhance the effectiveness of tumor treatments. However, Current methods to detect the therapeutic window for vascular normalization (histological/imaging techniques) face reproducibility and operational limitations, necessitating new biomarkers. Despite promising drugs, intrinsic/acquired resistance underscores the need to decipher vessel phenotypes and resistance mechanisms for personalized therapies. Recent clinical trials underscore the translational potential of vascular-targeting agents in pediatric thoracic tumors. For instance, bevacizumab, when combined with irinotecan, topotecan, or temozolomide in relapsed neuroblastoma, achieved a protocol-defined success criteria for overall response (complete or partial) rate and appeared to improve progression-free survival by normalizing aberrant vasculature and improving drug delivery (Moreno et al., 2024). Similarly, the multi-kinase inhibitor anlotinib reduced perfusion in pediatric soft tissue sarcomas, correlating with prolonged disease stabilization (Li, 2021). These findings highlight vascular modulation as a viable adjunct to cytotoxic regimens. By gaining deeper insights into these factors, we can develop more refined and personalized treatment strategies for patients, ultimately maximizing the chances of successful treatment outcomes for pediatric thoracic solid tumors.

The metabolic pathways of glycolysis and oxidative phosphorylation in tumor were highly complex and interconnected. For example, under the Warburg effect, tumor cells tend to favor glycolysis even when oxygen is abundant, yet oxidative phosphorylation within these cells is not entirely suppressed. The dynamic balance and switching mechanisms between these two pathways remain poorly understood. This complexity makes it challenging to precisely target metabolic pathways, as interventions aimed at a single metabolic step may trigger compensatory changes in other metabolic pathways. Such compensatory adaptations can undermine the effectiveness of targeted therapies, highlighting the need for a deeper understanding of these interconnected metabolic networks to develop more effective and specific therapeutic strategies. Metabolic interventions are gaining traction in pediatric oncology, with early-phase trials demonstrating feasibility. Notably, DCA restored mitochondrial respiration in neuroblastoma models, inhibits neuroblastoma growth by specifically acting against malignant undifferentiated cells—a critical advantage in pediatric populations (Vella et al., 2012). Amino acid and nucleic acid metabolic products play diverse roles in tumor. For instance, amino acids not only serve as building blocks for protein synthesis but also participate in cell signaling, energy metabolism, and various other processes. This multifunctionality complicates the development of drugs targeting amino acid metabolism, as inhibiting tumor cell growth may unintentionally affect other physiological processes. Genetic heterogeneity exists across different tumor types and even within the same tumor, leading to considerable variation in the reprogramming of amino acid and nucleic acid metabolism. For example, specific genetic mutations may alter the expression of amino acid transporters, thereby changing the intracellular amino acid metabolic state. This heterogeneity makes it nearly impossible to develop universal therapeutic strategies targeting amino acid and nucleic acid metabolism, necessitating personalized metabolic analysis and treatment design for each patient. Lipid metabolism encompasses the uptake, synthesis, and degradation of key lipid molecules, including fatty acids, phospholipids, and cholesterol, and these processes are continually dynamic in tumor. For instance, tumor cells can adapt the rates of lipid synthesis and degradation in response to nutrient availability and microenvironmental signals. This versatility and dynamism make it challenging to precisely identify the key regulatory points in lipid metabolism, which, in turn, complicates the development and application of targeted therapies aimed at lipid metabolism. The interplay between angiogenesis and metabolism offers untapped therapeutic synergies. Metabolic symbiosis induced by antiangiogenic therapy highlights cancer ability to evade therapeutic barriers by hijacking homeostatic mechanisms to fuel tumor evolution and undermine targeted treatments, dual inhibition of LDHA and VEGF receptors in murine models induced more tumor regression than monotherapy (Allen et al., 2016).

In summary, metabolic reprogramming encounters numerous challenges in the treatment of pediatric thoracic solid tumors. Although the metabolic pathways of pediatric thoracic solid tumors have been gradually discovered and continuously refined, most research has focused on tumor phenotypes rather than delving into downstream regulatory mechanisms. Given the complexity of metabolic mechanisms and the wide distribution of downstream targets for single regulatory factors, it is particularly important to identify the key metabolic dependency sites of tumors. It is hoped that future interdisciplinary approaches, utilizing multi-omics and organoid technologies, will provide new insights for targeted metabolic therapies in pediatric thoracic solid tumors, paving the way for the development of precision treatment strategies and improving patient prognosis.
